# Entropy and Fragility in Supercooling Liquids

**DOI:** 10.6028/jres.102.013

**Published:** 1997

**Authors:** C. A. Angell

**Affiliations:** Department of Chemistry, Arizona State University, Tempe, AZ 85287-1604

**Keywords:** energy landscapes, fragility, glass formers, glass transition, protein folding, relaxation

## Abstract

We review the Kauzmann paradox and what it implies about the configuration space energy hypersurface for “structural glassformers.” With this background, we then show how the relaxation expression of Adam and Gibbs qualitatively accounts for most of the phenomenology of liquid and polymeric glassformers including the strong/fragile liquid pattern, and the behavior of non-ergodic systems. Extended temperature range relaxation studies are consistent with a relaxation time pre-exponent on the quasi-lattice vibration time scale. When this boundary condition is imposed on Vogel-Fulcher-Tammann fittings, correspondence of *T*_0_ with *T*_K_ is found for liquids with *T*_g_ ranging over 1000 K. When it is imposed on the WLF equation *C*_1_ is obliged to become ~16, and the corresponding force-fitted *C*_2_ provides a measure of the polymer fragility which is generally not available from thermodynamic studies. Systems which exhibit discontinuous changes in configurational entropy on temperature increase, which include unfolding proteins, are briefly reviewed.

## 1. Introduction

**Fig. 1:**
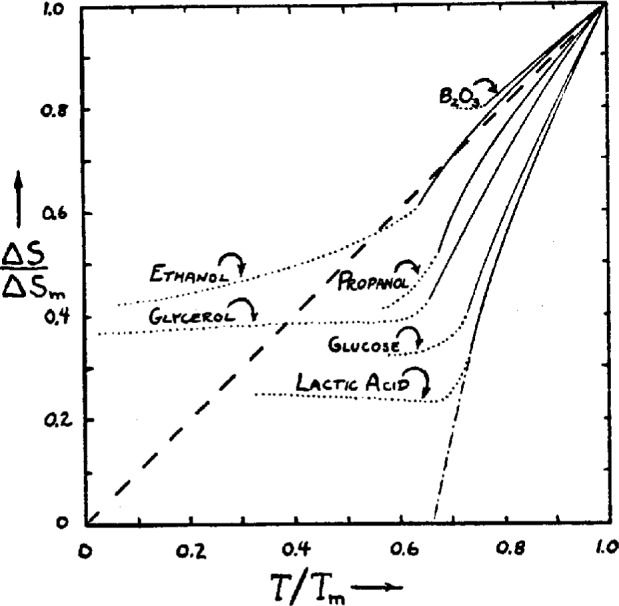
Kauzmann’s presentation of the entropy crisis which bears his name. The figure shows the rate at which the difference in entropy between liquid and crystal, normalized at the fusion point, disappears at *T* is lowered towards absolute zero. For B_2_O_3_, now known as a “strong liquid,” the liquid would always be of higher entropy than the crystal, even if the glass transition did not intervene at high *T*/*T*_m_, to change the heat capacity. At the other extreme, lactic acid loses its excess entropy so rapidly on cooling that if *T*_g_ did not intervene to arrest the loss, liquid would arrive at the same entropy as the crystal at 2/3 of the melting point. (This is the temperature usually associated with the temperature of the glass transition itself (the 2/3 rule which this set of data only weakly support).) Lactic acid is an example of a “fragile” liquid. Other examples of these plots for fragile liquids are given in Ref. [2].

Walter Kauzmann wrote only one paper on the subject of supercooling liquids and glasses, but it has proved to be one of the most influential papers in all of glass science [1]. There are many ways of presenting the main point he sought to make, but none are more telling than the graphical presentation chosen by Kauzmann himself. Thus we reproduce in Fig. 1 the key figure from this seminal paper. We find this presentation particularly attractive because it combines the key point which Kauzmann wanted to make, with a demonstration of the concept of fragility [2] to which we have given much attention in our own work of the past decade. We will briefly review the paradox presented by the data contained in Fig. 1, and then devote the remainder of this article to the manner in which the approach of Gibbs and his coworkers to this intriguing problem leads to an effective (though so far inexact) resolution of the paradox, and at the same time, to a very broad qualitative understanding of the phenomenology of viscous liquids and the glasses which form from them.

## 2. The Kauzmann Paradox and the Potential Energy Hypersurface for Glassformers

In Fig. 1, the difference in entropy between the crystal and liquid at the melting point, Δ*S*_f_, is used as a scaling parameter to permit the simultaneous comparative display of the manner in which the difference in entropy between the liquid and crystal states, for six different substances, varies during their supercooling. All cases show positive slopes, reflecting simply the fact that liquids have higher heat capacities than the corresponding crystals. What is interesting is the magnitude of the slope.

In the case of boron trioxide, now much spoken of as a “strong” liquid [2a,2c,^3^,^4^], the slope is such that the excess entropy of the liquid over crystal is only tending to disappear in the vicinity of 0 K—which raises no concern at all. On the other hand, to various degrees, the other liquids in the figure show provocative behavior. In the case of lactic acid, which we would now call the most “fragile” of the six, the excess heat capacity of the liquid over crystal is causing the excess entropy to decrease so quickly that a simple extension of the behavior would lead that excess to vanish at a temperature which is only ~2/3 of the fusion temperature. As far as can be told from the data, all that prevents this at first sight mind-boggling thermodynamic inversion from occurring, is the occurrence of a *kinetic* phenomenon, the glass transition at the temperature *T*_g_. At *T*_g_, the increasingly sluggish motion of the particles prevents further configurational changes to more ordered states from occurring as cooling proceeds at a fixed rate, and thus prevents equilibrium from being attained at lower temperatures. The consequent decrease in heat capacity, which is the signature of the glass transition, means the rate of entropy loss from the liquid is decreased, and the crisis is averted, as indicated in the diagram. The avoiding of a thermodynamic crisis by intercession of a purely kinetic phenomenon constitutes a paradox, the Kauzmann paradox, which must be resolved before glass science can be considered to have reached the state of a “mature field” of research.

Two quite profound theoretical problems are presented by the data of Fig. 1. The first is the problem of constructing an equilibrium theory for the liquid state that contains an explanation of how, on infinite time scales, the system evolves so as to undergo a rather abrupt, if not singular, change in heat capacity at some temperature between the glass transition temperature and absolute zero. Part of this problem involves the interpretation of fragility of liquids and the coupling of vibrational to configurational degrees of freedom. The second is the problem of constructing a theory which explains in a satisfying manner the reason why, in every case known, the kinetic characteristics of the liquid (which can to first approximation be represented by its diffusivity) evolve with temperature in such a way as always to generate equilibration times of the order of experimental time scales before the thermodynamic crisis is upon us.

Considerable headway on each of these problems was made by Gibbs and his coworkers [5,6,7] though they would be the first to recognize that the problems remained far from resolved by their work. Indeed, the “problem of glasses” remains a major challenge to the condensed matter theoretician and his experimental colleagues at this time [8].

Before reviewing the contributions of Gibbs et al., let us consider briefly what is implied about the topology of the chemical potential hypersurface which must be representative of substances exhibiting the type of behavior described above. Here we merely rephrase much of what was written by Gibbs and his contemporary Goldstein in articles written now some 25 years ago [9, 10].

The fact that glasses are brittle solids at temperatures below their glass transition temperatures implies that the arrangement of particles taken up as a liquid cools can be described by a point in configuration space near the bottom of a potential energy minimum in this space [10]. If this were not so, the system would move in the direction dictated by the collective unbalanced force acting on it, and some sort of flow would occur. Notwithstanding the myth about medieval cathedral windows [11], this does not occur in glassy systems held at temperatures less than half their glass transition temperatures, even on geological time scales. On the other hand, the existence of the annealing phenomenon, in which the density and energy of a glass formed during steady cooling can change with time on holding at a temperature below but close to the “glass transition temperature” means that there is more than one such mechanically stable minimum available to the system. Indeed, there would appear to be an almost infinite number, of order e*^N^*, where *N* is the number of particles in the system [12,13,14]. The minima obviously are distributed over a wide range of energies, the deeper minima usually being associated with higher densities. However, there are also many ways of organizing the same collection of particles into minima which differ negligibly in energy from one another.

**Fig. 2:**
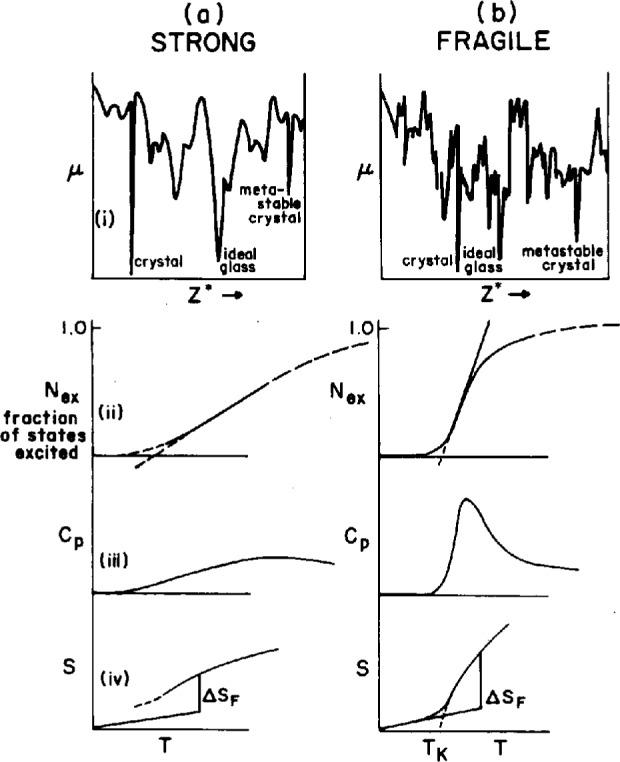
Sections through the 3*N* + 1 dimensional energy hypersurfaces of (a) strong and (b) fragile liquids. Z* is a collective configuration coordinate. Differences in the “density of minima” can be understood at an elementary level in terms of two-state models in which there are different increases in the number of distinct packings per elementary excitation event [16, 19] as represented in parts (ii), (iii), and (iv) of the figure for (ii) level of excitation, (iii) configurational heat capacity, and (iv) immediacy of the Kauzmann crisis, respectively, (from Ref. [2b] by permission). While this simple model clearly predicts, by extrapolation, an entropy crisis for fragile liquids at *T* > 0 K it has been shown that a kinetic arrest about 20 % above *T*_K_ does not satisfactorily resolve the Kauzmann paradox [16].

The fact that annealing proceeds more slowly the lower the temperature at which the annealing is carried out suggests that the process of finding deeper minima becomes more difficult statistically as the temperature is decreased. One arrives at the notion of an interconnected series of minima on a landscape of inconceivable complexity, in which increasing depth is coincident with decreasing population of minima, see Fig. 2 (from Ref. [2b]). The important implication of Kauzmann’s presentation in Fig. 1 is that for each system, at least for each “fragile” system, there must exist a statistically small number of minima at energies still well above that representing the crystal, and that these must set an absolute limit on the energy decrease achievable by annealing the amorphous system. It is into one of these last few minima that the system tends to settle at the temperature where the excess entropy tends to vanish. The temperature characteristic of this ground state for amorphous packing has become known as the Kauzmann temperature for that system, although the issue of the absolute value of the entropy, relative to that of the crystal that would be appropriate for a ground state system, remains unresolved. The issue is addressed in an important new paper by Speedy and Debenedetti [14] who succeed in evaluating quantitatively the number and distribution of minima (each of which they term “a glass”) for a model tetravalent system [15a, 15b]. This is an “inherent structure” [12] analysis which succeeds in providing a quantitative description of the liquid thermodynamics in terms of the inherent structure, and which appears to identify the density of the “ideal glass” for the model considered. The authors argue that this ideal state would be reached by a second order transition during slow (but not necessarily infinitely slow [14]) densification, and conclude it would have an entropy in excess of the crystal at the same *PV*_0_/*NkT*. Simple models of the two-state variety [16, 17] suggest the ideal glass would only be approached at 0 K, despite linear extrapolations which would indicate otherwise [See Fig. 2(ii)]. The existence, in principle, of an ideal glass state has been disputed by Stillinger [12, 18].

The more minima per unit of energy, the larger the configurational component of the total heat capacity should be, hence the larger the drop in *C*_p_ observed at the glass transition when ergodicity is broken during cooling. Of course, the change in *C*_p_ reflects the density of minima at the level of the landscape at which the system gets trapped during cooling, and this level will depend on the height of energy barriers separating the minima as well as the total degeneracy, as will be described in the next section. Both features of the landscape arise from the form of the interaction potential for the particles, but our knowledge of exactly how is in an elementary stage [12,13,14]. It is commonly found that fragile liquids, like the lactic acid of Fig. 1, have large changes in heat capacity at their *T*_g_, implying highly degenerate landscapes even quite close to *T*_K_.

It is simpler to discuss the potential energy hypersurface as opposed to the chemical potential (free energy) hypersurface, but it may also be less fruitful. For each interaction potential, there must exist a single immutable potential energy hypersurface for an *N* particle system; however, the free energy hypersurface will depend on temperature. While a hard sphere system will have a potential energy hypersurface that is totally degenerate—all configurations have zero energy—all configurations do not have the same *free* energy except at absolute zero. This is because the different configurations have different amounts of spare volume, and the *free* flight motion of spheres adjacent to such “loose spots” provides an entropy-generating mechanism. Thus different packings yield different pressures and different *free* energy minima. For the hard sphere system, these free energy minima have been evaluated by Dasgupta using a density functional approach [20]. Since we are most commonly interested in the behavior of systems at different temperatures, it seems that the most relevant hypersurface is the chemical potential hypersurface. In any case, both of these hypersurfaces are quite impossible to conceptualize given that they exist in a space of dimensionality of the order of the number of particles. The attempt to represent them by two-dimensional slices, such as illustrated in Fig. 2, is a grotesque, but frequently practiced, oversimplification. It is permitted by the community only because it provides a way of thinking about such problems as the annealing of glasses and the configurational entropy of disordered systems.

The individual minima on the free energy hypersurface are the configurational microstates, or “configurons” [21] of the system. Note that the configurational entropy of the system is related to the number of minima accessible (in the thermodynamic sense) to it at a given temperature, irrespective of whether the system has time to “explore” them all. Thus a system that has become confined to a single minimum (which we call a “glass”) still has an entropy in excess of the crystal, as shown in Fig. 1 by the bending over of the curves at the temperature *T*_g_. This is problematical because statistically a system confined to a single state should have unit probability apart from vibrations. This was recently considered by Bowles and Speedy [22] who elaborate on the distinction to be made between statistical entropy and thermodynamic entropy, and conclude that it is the latter, indicated in Fig. 1 by the positive entropy values below *T*_g_, which is important in determining equilibria between physical states.

While it is not obvious, the total number of configurons per *N*-particle system may be not very dependent on the nature of the interaction potential or even the organization of the particles into bound groups, i.e., molecules [15c]. Numbers roughly exponentiate with *N* [12–14], e.g., exp(1.2*N*) for the tetravalent model [14]. In this case the total entropy available by raising the temperature enough for all minima to be accessible, is *k*_B_ln*w*, hence about *k*_B_ln(exp*N*) i.e., about *R* entropy units per mole of particles. The “height” of the landscape will then be different for liquids of different fragility, because of the different changes of heat capacity Δ*C*_p_ they exhibit at *T*_g_. The quantity Δ*C*_p_ is the heat capacity increment due to gaining access to the configurational states, and it is its integral over the temperature range *T*_K_ – *T*_u_ which must amount to about *R* per mole of heavy atom centers. Thus *T*_u_, the temperature corresponding to the upper limit of the landscape (its “height”), will be high relative to *T*_g_ for strong liquids, which have small values of Δ*C*_p_. Thus the liquid state “stretches out” with decreasing fragility, and crossovers to free diffusion behavior will occur at higher temperatures, as seen in recent studies [3, 4]. This matter is treated in more detail in coming publications.

To be in equilibrium (except with respect to crystallization), a system must be able to visit, move between, a representative subset of the minima characterizing its chemical potential hypersurface. In this case, it is not correct to speak, as is often done, of a system well below its glass transition temperature as existing in any one of a manifold of “metastable states.” These are “mechanically stable” but not “metastable” states. When held at a temperature near but below the glass transition temperature established during normal cooling, the system “anneals” by exploring the lower energy minima which were inaccessible time-wise during the initial cooling. Since, for most potentials, these are minima in which the particles are more densely packed, the glass volume will usually decrease during annealing. Even at constant density, however, annealing can occur since, in a complex system, configurations of different energy may have the same volume. Equilibration is made simpler by the fact that a macroscopic system consists of statistically independent nanoscopic regions such that the mean distance moved, by a single particle during equilibration, is only a fraction of a molecular diameter [23].

Annealing occurs more slowly at lower temperatures for two reasons. Firstly there are energy barriers to be crossed in the process of passing from minimum to minimum—the system collectively vibrates for increasingly long periods before some chance fluctuation (associated with exceptionally anharmonic excursions on the parts of some particles) permits it to rearrange, and hence to enter a new minimum. Secondly, there are entropic barriers because the lower energy minima are more distantly spaced, hence the number of successive rearrangements which must be made in order to arrive at one of the lower energy states must increase as temperature decreases. This aspect of relaxation has been considered recently by Mohanty et al. [24]. The combination of barriers would seem to ensure that no matter how slowly a liquid is cooled (or equivalently how long a glass is annealed), the system will never reach the isoentropy condition in finite times (although *T*_g_ itself might appear to become independent of cooling rate [25]). Thus *T*_K_ for the “configuron bath,” like absolute zero for the “phonon bath,” is inaccessible.

This line of thought was made more quantitative by the development of the “entropy theory” of Adam and Gibbs [7], the usefulness of which we now discuss in some detail.

## 3. Relaxation and Entropy

At the time that the Adam-Gibbs equation was written down, it was very popular to explain the slow dynamics of supercooled liquids, and particularly of chain polymers, in terms of the free volume concept. The idea that a liquid’s particles, or a polymer’s segments, move around at rates proportional to the amount of “elbow room” they find available, was both simple and satisfying, and the free volume concept was found to provide a convincing rationale of such important equations as the WLF equation [26] which described the temperature dependence of relaxation times in a wide variety of polymeric liquids. The Adam-Gibbs theory, which was based on a modification of conventional transition state theory to accommodate the notion that, in viscous liquids, the rearrangements over energy barriers must be cooperative and, further, that the size of the cooperating groups would necessarily increase with decreasing temperature, was running against the tide and was given little attention. Twenty-five years later, we see it in the ascendancy.

The Adam-Gibbs approach led to an expression for the relaxation time which contains the excess (configurational) entropy, *S*_c_ of the Kauzmann paradox, in the denominator of the exponent, a result of an inverse relation between z*, the minimum size group of “beads” (rearrangeable units) capable of undergoing a rearrangement at temperature *T*, and the configurational entropy *S*_c_. The expression is

$$\tau =\tau_{0}\exp\left(\frac{C\prime \Delta \mu }{TS_{c}}\right)$$ (1)

where Δ*μ* is the conventional free energy barrier (per molecule in the cooperative group) to rearrangements, and *C*′ is a constant. The familiar departure from Arrhenius behavior comes from the temperature dependence of Sc which itself depends on the value of the configurational heat capacity. This is manifested at the glass transition by the change in heat capacity Δ*C*_p_.

**Fig. 3:**
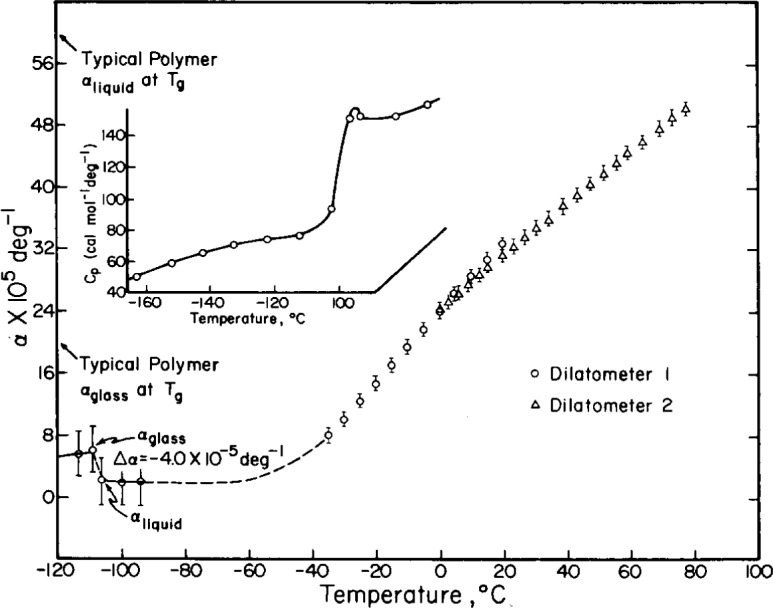
Expansion coefficients for the liquid and glassy states of lithium acetate + water solution of mole ratio 1:10, as a function of temperature. Inset: a typical DSC trace obtained from a LiOAc · 10H2O sample showing the large jump in heat capacity at the same *T*, where the small decrease in *α* occurs.

At the time of its publication, the Adam Gibbs approach was very appealing to the present author because of the interesting behavior observed in some aqueous solutions under study at that time [27]. We encountered cases, such as that illustrated in Fig. 3, in which the glass transition temperature was associated with a zero or slightly negative change of expansion coefficient, Δ*α*. Consistent with the second Davies-Jones relation [28],

$$dT_{g}/dp=VT\Delta \alpha /\Delta C_{p},$$ (2)

these systems showed negative slopes of *T*_g_ vs *P*, in striking contrast to the behavior of polymers and most simple liquids. Free volume concepts clearly do not make much sense for such systems, yet the same solutions showed large increases in heat capacity at *T*_g_ (see Fig. 3), and hence behaved in a manner perfectly consistent with the Adam-Gibbs equation. Thus the Adam-Gibbs approach would appear to be more fundamental than the free volume approach since it applies irrespective of whether or not entropy fluctuations are correlated directly or inversely (the cases of supercooled water, silicon, and SiO_2_, which all have density maxima below their *T*_m_) with volume fluctuations [29].

We will not discuss the derivation of Eq. (1) except to note the general view of theoreticians that it cannot be understood (see, e.g., the contribution of E. A. Dimarzio in this volume). Notwithstanding this view, the equation appears to contain a lot of truth since it has excellent predictive capabilities. We will consider these capabilities under three conditions: a) relaxation very far from equilibrium, i.e., in the glass when the configurational energy is essentially constant; b) relaxation in the ergodic domain above *T*_g_; and c) relaxation at temperatures near *T*_g_ where the glass is annealing.

### 3.1 Relaxation in the Non-Ergodic State

For a fixed value of the configurational entropy, Eq. (1) predicts that such relaxations as may be observed, will have Arrhenius character, the slope of the Arrhenius plot being inversely proportional to the value of the entropy held constant.

Evidence for the essential correctness of this prediction can be obtained from different sources. The first example is the Arrhenius variation of the electrical conductivity of ionic glasses. Well above *T*_g_, the ionic inverse conductivity follows the viscosity in its tendency to diverge near the Kauzmann temperature [30, 31]. However close to *T*_g_ it tends to decouple, assuming a smaller temperature dependence, and then finally changes slope again at *T*_g_ to assume its glassy state value [32]. As Eq. (1) predicts, the Arrhenius activation energy is proportional to the amount of annealing which has been imposed on the glass since this lowers *S*_c_. The smaller *S*_c_ becomes, the larger the glassy state activation energy. This correlation is more pronounced the more closely the conductivity is coupled to the viscosity [33].

**Fig. 4:**
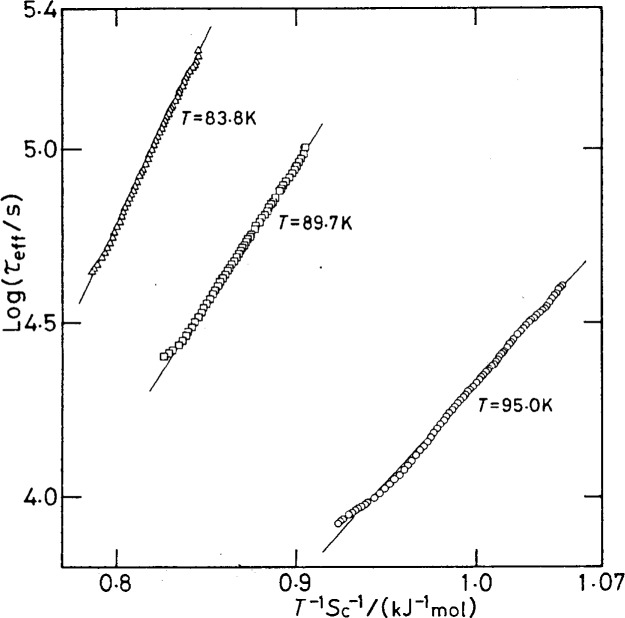
Test of the Adam-Gibbs equation for the relaxation time of butyronitrile vapor-deposited samples (from Ref. [34] by permission).

A more direct example is offered by studies of vapor-quenched glasses of high excess entropy [34]. These can be studied at different entropy levels, fixed by annealing the deposits for different annealing times at higher temperatures. Equation (1) then predicts that, when the temperature is chosen to bring the relaxation into the experimental time window, the relaxation time (which can be obtained from sensitive calorimetric studies at the lower temperatures [28]) will be a linear function of the inverse product *TS*_c_. The validity is demonstrated in Fig. 4 [34]. As predicted, the slope is larger (and *τ* is longer) the smaller the average value of *S*_c_.

### 3.2 Relaxation in the Ergodic Domain

#### 3.2.1 Liquids

**Fig. 5:**
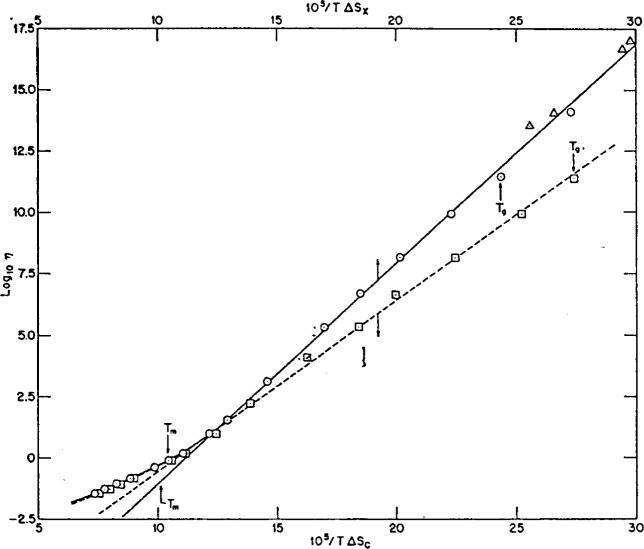
Test of the Adam-Gibbs equation for viscosity of tri-napthyl benzene at temperatures above *T*_g_. *S*_c_ has been assessed in two different ways leading to two different plots (see text), each of which is seen to be linear over a wide range of the variable (*TS*_c_)^−1^ c (from McGill, Ref. [35] by permission). (Δ*S*_c_ in the paper of Magill is the *S*_c_ of this paper.)

Most of the tests of Eq. (1) have been carried out in the region of temperature above *T*_g_ where the value of *S*_c_ is an equilibrium quantity and one which changes systematically with the temperature. Eq. (1) predicts that under these circumstances, there will only be a single relaxation time vs temperature relation and that it will be a linear one for log*τ* vs (*TS_c_*)^−1^. An example is given in Fig. 5 for the case of tri alpha naphthyl benzene [35], for which the configurational entropy was obtained from differential scanning calorimetry. Log (viscosity) is seen to be linear in (*TS_c_*)^−1^, at low temperatures, and the curvature at high temperatures is as likely to be due to uncertainties in the assessment of *S*_c_ [taken as either the difference between liquid and crystal entropies, ignoring differences in vibrational entropy at high *T*, (upper curve) or between liquid and glass at *T*_g_ (lower curve)] as it is to failure of the theory.

A more frequently used, though not as direct, test of the Adam-Gibbs equation is to develop the equation into a *T*_0_ Vogel-Fulcher-like form and then demonstrate that the *T*_0_ parameter has a value close to that of the Kauzmann temperature obtained by the purely thermodynamic route. In their original treatment, Adam and Gibbs made the simplest assumption for the excess heat capacity, which determines the configurational entropy temperature dependence, viz., that it is a constant. This then yields

$$S_{c}=\Delta C_{p}\ln T/T_{K}.$$ (3)

Substitution into Eq. (1) then yielded the Vogel-Fulcher equation as an approximation, valid near *T_K_*. However Δ*C*_p_ = constant does not describe many molecular systems. More accurate [36, 37] is Δ*C*_p_ = *K*/*T*, from which

$$\Delta S(T)=K(T–T_{K})/TT_{K}$$ (4)

which leads to the Vogel-Fulcher equation as an identity

$$\tau =\tau_{o}\exp DT_{0}/(T-T_{0})=\tau_{0}\exp(-F\varepsilon )$$ (5)

where *F* is a fragility parameter, 0 < *F* < 1, and *ε* = (*T*/*T*_0_ − 1), with *T*_0_ = *T*_K_.

There has always been dispute concerning the validity of Eq. (5), and this has been revived with vigor recently in the light of a temperature derivative analysis by Stickel et al. [38, 39]. Stickel et al. show that, particularly for fragile liquids, there is a region at relatively high temperatures where the *V*–*F* equation fits quite well but yields a *T*_0_ that is considerably higher than *T*_K_ and often is also larger than *T*_g_ (a result which is unphysical and is associated with unphysical values of the *τ*_0_ parameter). This analysis emphasizes the high temperature data whereas an analysis focusing on the last five to six decades in relaxation time (covering a small range of ordinate values on the Stickel plot) yields a lower value of *T*_0_, one which usually agrees rather well with the Kauzmann temperature, while also yielding a physical (phonon-like) pre-exponential *τ*_o_. We will document this below for a large number of different glassformers. For intermediate liquids such as glycerol, even the Stickel analysis yields a *T*_0_ in good accord with *T*_K_ [38].

In Table 1, we record *T*_g_, *T*_K_ and *T*_0_ data for a compendium of liquids of different classes which have been studied over the years. The best fit values of *T*_0_ are reported for two different temperature regions relative to the glass transition temperature, where the data are available. When *T*_0_ depends on temperature, the value which should be compared with *T*_K_ is the one associated with the most physical pre-exponent, e.g., *τ*_0_ = 10^−14^ s. This value is the inverse of the phonon frequency (*f*(Hz) = 1/2*πτ* = 2 × 10^13^ Hz) typifying the liquid quasi-lattice (which should set the attempt frequency for cooperative rearrangements). It is also the value of the pre-exponent found for relaxation in plastic crystals with very “strong” character (Arrhenius behavior) in which the extrapolation to 1/*T* = 0 is unambiguous [40].

The best fit *τ*_0_ value, going with the value of *T*_0_ used in the final column comparison in Table 1, is listed in the second final column so that the physical content of the *T*_K_/*T*_0_ comparison can be judged. If the number in this column is larger than 14, it suggests that the value of *T*_0_ used in the comparison with *T*_K_, although best-fitting the relaxation data, may be inappropriately low for the comparison. The values of the ratio *T*_K_/*T*_0_ would be unity if Eq. (1) properly described the liquid behaviour over wide temperature ranges, and if the basis for obtaining Eq. (5) from Eq. (2) is applicable. We note that in many cases of higher *T*_g_ systems, Δ*C*_p_ is not hyperbolic in *T*_g_, in which case Eq. (5) should only hold approximately near *T*_g_.

Table 1 shows that *T*_K_/*T*_0_ values close to unity are obtained for glassformers with *T*_g_ varying between 50 K and 1000 K. Since the liquids represented in Table 1 range from molecular through covalent (Se, As_2_Se_3_) to complex ionic oxides, we judge the case for the Adam-Gibbs approach to relaxation in glassforming liquids to be a strong one.

An objection often raised to the Adam-Gibbs theory, and other approaches that similarly suggest that a thermodynamic phase transition underlies the glass transition, is that no experiment has detected a diverging length scale, *ξ*. However, Eq. (1), with *S*_c_ ~1/z* ~1/(ξ*)^3^ together with Fig. 1, shows why a diverging length scale would be extremely difficult to detect. Since *S*_c_, even in the most fragile cases studied to date, rarely falls to less than one-third the entropy of fusion (Fig. 1, Refs. 1–30 in Table 1), it must be expected that (z*)^−1^ will only decrease to one-third of its initial value as *T* decreases from *T*_m_ to *T*_g_. If *ξ* (~z*^1/3^) is of molecular dimensions, say 6 Å at *T*_m_, then according to Adam and Gibbs, the characteristic length would only increase from 6 Å to 6 × (1/3)^−3^, i.e., by ~2 Å over this whole *T*_m_ to *T*_g_ range. Thus the Adam-Gibbs theory predicts that, even though ξ should diverge as *T* → *T*_K_, identifying a changing length scale by computer simulations, which can only probe much smaller decreases of *S*_c_ than the above, will be an unrewarding endeavor—as indeed has been found [41].

#### 3.2.2 Polymers

Since many studies have demonstrated a relationship between the different canonical characteristics of relaxing liquids, and since aging (which is simply a slow and unwelcome approach to equilibrium at *T* < *T*_g_) is a considerable problem in polymers, it is important to have some way of estimating how far above the ground state temperature a given polymer system is at ambient temperature. The problem with polymers is that reliable estimates of the Kauzmann temperature are frequently not available because of the failure of the system to register a clean crystallization, hence a quantifiable entropy of fusion. However, if the Adam-Gibbs equation is as good as it appears from Table 1, then a reliable value of *T*_0_ would serve the purpose. We show how to achieve this in the following analysis.

A great deal of relaxation data for polymers have been analyzed using the Williams-Landel-Ferry equation,

$$\log\tau /\tau_{g}=C_{1}(T-T_{g})/[T-(T_{g}-C_{2})].$$ (6)

It is well-known that the Williams-Landel-Ferry and Vogel-Fulcher equations are mathematically equivalent. In the simplest case in which both are valid at all temperatures, values of *T*_K_ would be available from the relation *C*_2_ = *T*_g_ – *T*_0_ assuming from before that *T*_0_ ≡ *T*_K_. However, just as the Vogel-Fulcher equation usually does not provide an adequate fit of the data over the whole range of possible relaxation times, so does the WLF equation provide parameters which are frequently inapplicable outside the range of data taken, hence which are unreliable guides to the Kauzmann temperature. As seen above (Table 1), for applications of the Vogel-Fulcher equation to molecular liquids, *T*_0_ is found to lie close to *T*_K_ when the pre-exponent, *τ*0, of Eq. (1) lies close to 10^−14^ s, which is the physically meaningful value for barrier crossing (or de-trapping) processes. We have shown elsewhere [42] that for the case *τ*_0_ = 10^−14^ s, *C*_1_ of the WLF equation should have the value 16 because of the relationship (which is not generally to be found in textbooks),

$$C_{1}=\log\tau_{g}/\tau_{0}.$$ (7)

**Fig. 6:**
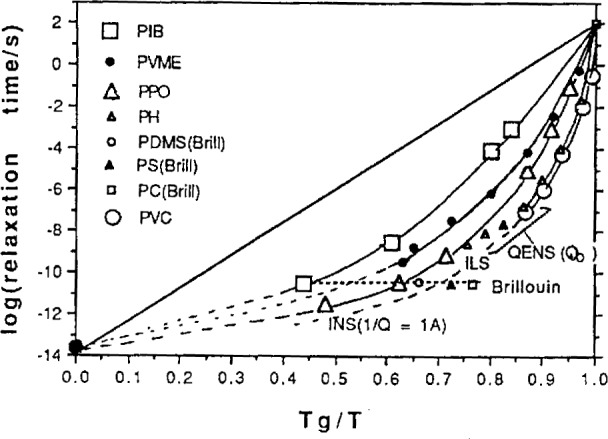
*T*_g_-Scaled Arrhenius plots of segmental relaxation times for linear chain polymers based on mechanical relaxation, light scattering, and NMR ^13^C segmental relaxation time data. *T*_g_ is defined by the temperature at which *τ* = 10^2^ s. The data extrapolations suggest an infinite temperature value of about 10^−14^ s, consistent with Raman modes associated with short wavelength acoustic phonons which, in the absence of selection rules, dominate the Raman spectrum in this frequency range ● [43]. The identifications of data sources on the diagram are as follows: INS = inelastic neutron scattering for the inverse wave vector, *Q*^−1^ = 1 Å; [44] QUENS (*Q*_0_) = Quasielestic neutron scattering at peak of structure factor, *Q*_0_; [45] ILS = impulsive light scattering [46].

For cases with *τ*_o_ = 10^−14^ s, the value *C*_1_ = 16 follows from the definition *τ*_g_ = 10^2^ s. Thus for cases in which the WLF parameters include *C*_1_ ~ 16, the ground state temperature *T*_K_ can be obtained as *T*_g_ – *C*_2_. Likewise the fragility can be taken as

$$F=1-C_{2}/T_{g}=T_{o}/T_{g}=T_{o}/T_{g}$$ (8)

(where *T*_o_ is the Vogel temperature) since this provides a number which varies between zero and one with increasing fragility. The most fragile of the chain polymers, according to various studies [47] is polyvinyl chloride (PVC) for which the long-established WLF parameters are *C*_1_ = 16.2, *C*_2_ = 25 K [48]. Since *C*_1_ has the physical value, *T*_K_ can be obtained from *C*_2_ and *T*_g_. The fragility accessed by the above expression, Eq. (7), is indeed close to unity, 1 – *C*_2_/*T*_g_ = 0.85. By contrast, polyisobutylene (PIB) has *C*_1_ = 16.5, *C*_2_ = 104, and *F* = 1 – *C*_2_/*T*g = 0.48. The general consistency of chain polymers with the strong/fragile pattern, and with τ_o_ values of the order of the vibration period is shown by the selection of data in Fig. 6.

### 3.3 Relaxation in the Non-Ergodic State Near *T*_g_

**Fig. 7:**
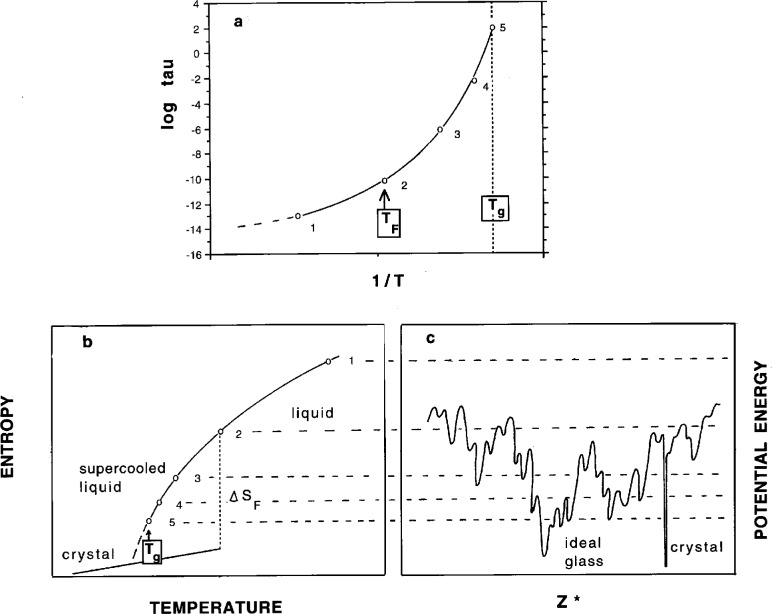
Illustration of the relation between relaxation time, entropy, and excitation level on the potential energy hypersurface for a fragile glassforming system. Point 1 is in the free diffusion regime, unperturbed by any barriers to cross or traps to escape from. This is the regime of mode coupling theory validity. Around point 2, the melting point of this model glassformer, the system begins to “sense” the landscape, and it becomes increasingly enmeshed as *T* → *T*_g_ at point 5. With relatively little excess entropy (the “lifeblood of the liquid state”) remaining, the system falls out of equilibrium, becoming trapped in a single minimum (becomes a “glass”) as its relaxation time rapidly increases beyond the normal experiment measurement timescales. The ideal glass is the configuration shich has energy within the lowest well of all (excluding crystal wells), which would be occupied at *T*_K_ in a sufficiently slow cooling process. Then the configurational component of the total entropy would be *k*_B_ln1 = 0.

Near *T*_g_, relaxation is complex because the quantity *S*_c_ of Eq. (1) is time-dependent. Thus a measurement performed at a constant temperature will yield a relaxation time for recovery of the equilibrium state which is never a linear function of the displacement because *S*_c_ itself relaxes according to an Adam-Gibbs equation. When *S*_c_ finally reaches its equilibrium value, the relaxation time, of course, becomes time-independent. Behavior in this region has been treated in detail in the review by Hodge [49] and is additionally covered by the contribution of Hodge in this symposium issue. Here we note only that if the temperature of the isotherm falls below *T*_K_, then the relaxation time will necessarily diverge as waiting time increases. A diagram summarizing the relation between the accessible microstates, relaxation time and configurational entropy is given in Fig. 7.

## 4. Systems With Discontinuous Changes in Configurational Energy and Entropy

In a minority of cases, mainly those with tetrahedrally coordinated, inefficiently packed, ground configurational states, discontinuous changes in configurational entropy, hence in relaxation time, seem to be possible. In the laboratory liquids so far discussed, this phenomenon appears to occur in the supercooled state in association with high crystallization rates, so the phenomenon is not easily studied. Furthermore, it is often not possible to be sure that the change is discontinuous (first order) rather than continuous through a smoothed higher order transition [50]. The clearest case which has been documented by computer simulation studies [51] as well as circumstantially by laboratory studies [52] is liquid silicon. The case of water is controversial [50,53,54,55,56,57]. Current studies [58] suggest that a similar phenomenon should occur in liquid beryllium fluoride at temperatures above the melting point (but also, unfortunately, above the normal vaporization temperature). It is hoped that, by control of pressure, some direct observations of the phase transition in the latter system may become possible.

**Fig. 8:**
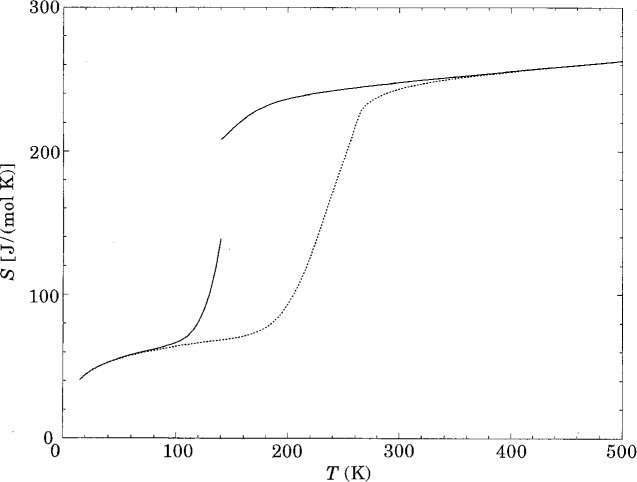
Discontinuity in entropy, and precursor effects at a liquid-liquid transition obtained at ambient pressure in one parameterization of the Poole model for water-like substances. This can occur when the hypersurface is characterized by the two megabasins [2c] separated by a substantial energy barrier. The megabasins usually seem to differ in topology, the high density one being characteristic of a fragile liquid, and the low density one, strong. (Adapted from Ref. [68].) Dotted line shows behavior for parameterization with stronger bonds.

An important finding associated with these transitions, whether first order or continuous, is that the character of the liquid changes abruptly on approach to the fully coordinated state and that the change is one from fragile to strong liquid behavior. This interesting phenomenology is in the early stages of its exploration. An account of the origin of the phenomenon in its different possible manifestations is given in the thermodynamic model of Poole et al. [59] and in the several microscopic models by Debenedetti and coworkers [60,61,62,63]. The entropy discontinuity, according to one parameterization of the Poole model, is shown in Fig. 8.

There is an interesting possibility that the same phenomenology might be available in mesoscopic systems of the biopolymer variety. Evidence presented elsewhere [2c] suggests that when proteins unfold, there is a change in character from strong liquid to fragile liquid. Since in these cases there is no interference from crystallization phenomena, there is some hope that systematic studies of this provocative phenomenology might be made with such systems or with model systems based on them. Not only proteins behave in this manner but so do various RNA molecules. The attempt to correlate this aspect of biophysics (or, better, biopolymer physics) with the phenomenon of polyamorphism in molecular systems, is in its infancy, but it bears directly on such important societal problems as “mad cow” disease, which involves refolding of “good” proteins into lethal polyamorphic forms [64,65,66]. This relation is discussed in more detail elsewhere [67].

## Figures and Tables

**Table 1:** Correlation of Kauzmann temperatures *T*_K_ with Vogel-Fulcher *T*_0_ to values for various substances

Substance	*T*_g_^a^	*T*_K_	Ref.^b^	*T*_0_ (high)	*T*_0_ (low)	Ref.	*T*_g_/*T*_0_	*T*_g_/*T*_K_	frag. *m*^c^	(frag.)^−1^ *D*	−log*τ*_0_^d^ (−log*η*0) (−log*D*_0_)	*T*_K_/*T*_0_
1-butene	58^a^	48	1	64(*η*)	[54(*η*)]	31	1.07	1.20				>0.88
2-methylpentane	78^a^	58	2	59(η)	60+5 (η)	32, 33	1.43	1.38	58			0.97
	83			n-hex.								
butyronitrile	100	81.2	3		58(τ_D_)	34	1.72	1.19	47	32	16	1.26↓
ethanol	95	71	4b		70–75(τ_D_)	35	1.28	1.33			7	1.0
	90^a^			80(τ_D_)		36	1.19			2.7	8.9	0.93↑
methanol	103	64±5	5, 6		60±15(*τ*_D_)	37(a)	1.71	1.61		12.4		1.06
			9	66(D)		37(b)				12.4	(7.1)	0.97
n-propanol	105	73	7	73.5(η)	73.5(*τ*_D_)	38	1.42	1.44	33		11.7	1.00↑
	100^a^				50.3(*τ*_D_)	36			40		12.4	1.45
toluene tol + 17% benzCl	126	96	8	103(η)	<108(*τ*_D_)	39(a)	>1.16	1.19		5.6	(3.5)	0.93
	126			108(*τ*_D_)	108(*τ*_D_)	39(b)	>1.16		107		13.0	~1.0
ethylene glycol (ethan diol)	153	115	9		109(*τ*_D_)	40	1.43	1.33		16	14.3	1.05
		119	10	125 (η)		41	1.22					
1–3 prop. diol	154	109	10					1.41				
	145^a^											
1–2 prop diol (*T*_g_=*T*_g_(1·3)+18	172	109+18?	11	109(η)	114(τ_H_)	42	1.57	1.41	52	17.8	14.6	1.11
		(127)			122(τ_D_)	42			52	13.5	13.2	1.04
glycerol	193	135	12b		128(τ_H_)	42	1.51	1.43	53	19.5	15.6	1.04
	187				137(τ_H_)	44	1.41		53			0.99
					127(τ_D_)	38,9	1.52			12.7	14.6	1.07
					127(*τ*_D_)	45				8	33	1.07↓
				121(*τ*_D_)		45(b)						1.11
H_2_SO_4_·1H_2_O	182	142	13b	146(σ)		46	1.25	1.28				0.97
H_2_SO_4_·2H_2_O	169	131	13b	120(σ)		46	1.41	1.29				1.09
H_2_SO_4_·3H_2_O	162	135	13b	128(σ)		46	1.27	1.20				1.05
	155^a^											
H_2_SO_4_·4H_2_O	157	133	13b	136(η)		46	1.15	1.18				
				136(σ)		46						0.98
triphen.phosfite	205	166	14	183	186	34	1.12	1.23	160	2.9	13.3	0.91
PMS (disiloxane)	165^a^	137	15(a)			47		1.20				
Salol	220^a^	167	16		135(τ_H_)	48	1.63	1.31		38.7	24.5	1.24↓
		157	17,48		141(τ_D_)	48	1.56	1.40		33	23.3	1.11↓
orthoterphenyl and otp + 16% opp	244	200	18		184(τ_H_)	49(a)	1.33	1.22			17.7	1.09↓
					196(η)	17, 32	1.26	1.26	81^c^			1.04
					193(η)	50						
dibutyl phthallate1 (uncrystallizable)	179		19	151(η)	151(η)	51	1.18				(3.6)	
					137(*τ*_D_)	32, 49(b)	1.30		69		14	
*m*-toluidinc	187	151	14,8(b)		153(*τ*_D_)	8(b)			79		13	1.00
		157	15(b)									
propylene carbonate	156	125.8	20		130(τ_D_)	52	1.20	1.23	104	2.9	13.1	0.97
		128	52b		132.3(*τ*_D_)	36						0.95
Ca(NO_3_)_2_·4H_2_O	217	200	21	205(η)		53		1.09				0.98
		204	22		190(*η*)	54	1.14					1.05
				201(*σ*)		55	1.08					1.0
Cd(NO_3_)_2_·4H_2_O	213	198	21			56		1.08				
fructose	286	210	14		206(τ_E_)	57	1.39	1.36			13.5	1.02
glucose	306	271^a^	23		231	58	1.32	1.13				1.17
					259(*τ*_D_)	43		1.18			12	1.05
mannitol	282	236	9, 24									
sorbitol (dulcitol)	266	236?	24	212(η)		59		1.13	93	8.6		1.11
		217	24		224(τ_D_)	9	1.19	1.23		7.8	14.3	1.05
sucrose	323	283	25		290	58	1.11	1.14		0.154		0.98
		287	14					1.125				
trehalose	388		14			57					13.5	
phenolphtalein	363	310	14		274(τ_E_)	57	1.32	1.17			13.5	1.13
selenium	307	240±10	26		251(η)	32,63	1.22	1.28	87^c^			1.04
ZnCl_2_	380	250±25	27	260(η)		60	1.46	1.52				0.96
					180(*τ*_1_)	32,64			30^c^		14	1.39
					236(τ_V_)	32,65	1.61		42.5^c^		14	1.06
Li acetate	401	381^e^	28	371(σ)		61	1.08	1.05^a^			14	1.03
As_2_S_3_	455	265	26		237(τ_h_)	44	1.82	1.93			18.7	1.00↓
La_2_O·2B_2_O_3_	959	845	29	864	850(η)	29	1.12	1.13				0.99
CaAl_2_Si_2_O_8_	1118	815	30		805(η)	62	1.39	1.37				1.01

a *T*_g_ value based on the onset *C*_p_ from adiabatic calorimetry, which is several degrees lower than scanning calorimetry or DTA-based values because of the much longer time scale. *T*_g_/*T*_K_ values are based on 10 K/min DSC or DTA data for *T*_g_.

b Indicates that the assessment of *T*_K_ will not be found in the calorimetry paper cited, but rather in one of the authors articles, or students’ thesis.
C. A. Angell and W. Sichina, Ann. N.Y. Acad. Sci. Vol. 279 (1976) p. 53. E. J. Sare, Ph. D. thesis, Purdue Univ. (1970). D. L. Smith, Ph. D. thesis, Purdue Univ. (1983).

c Many values of *m*, defined as the slope of a Fig. 6 type plot at *T*_g_/*T* = 1, are compiled in R. Bohmer, K. L. Ngai, C. A. Angell, and D. J. Plazek, J. Chem. Phys. **99** (5), 4201–4209 (1993). Where the *m* value is used to obtain *T*_0_ via Ref. 32, the superscript c is attached to the *m* value. Such *T*_0_ values are associated with −log*τ*_0_ of 14 by assignment.

d −logτ_0_ is the value of τ_0_ which is the best fit value for the *T*_0_ value cited. If −log*τ*_0_ is numerically larger than the physical value of 14 (phonons), then the *T*_0_ value should be weighted up, and therfore *T*_K_/*T*_0_ should be weighted down. Where this is an important effect, the value of *T*_K_/*T*_0_ is tagged ↑ or ↓ to indicate the need for adjustment. For viscosity, the equivalent value of −log(*η*_0_/*P*) is 3.5 and for diffusivity −log(*D*_0_/m^2^s^−1^) is 7.55. For cases in which *T*_0_ is obtained from an *m* value via Ref. 32, the value of −log*τ*_0_ is 14 by assignment.

e Unpublished data (Sichina and Angell) suggest this estimate of *T*_K_ is too high, that Δ*C*_p_ passes through a maximum and *T*_K_ retreats to ~ 360 K.
*σ* *T*_0_ value from conductivity. *η* *T*_0_ value from viscosity measurements. *τ*_D_ *T*_0_ value from dielectric relaxation measurements. *τ*_E_ *T*_0_ value from tensile stress relaxation measurements and assignment *τ*0 = 10^–13.5^ s. *τ*_H_ *T*_0_ value from ac heat capacity measurements. *τ*_L_ *T*_0_ value from longitudinal relaxation time from digital correlation spectroscopy. *τ*_h_ *T*_0_ value from Sherer-Hodge *T*_g_ analysis Ref. 44. *τ*_V_ *T*_0_ value from volume relaxation activation energy at *T*_g_ and Ref. 32.

References to Table

1 J. G. Aston, H. L. Fink, A. B. Bestul, E. L. Pace, and G. J. Szaca, J. Am. Chem. Soc. 68, 52 (1946); S. S. Todd and G. J. Parks, J. Am. Chem. Soc. 50, 1427 (1928).

2 D. R. Douslin and H. M. Huffmann, J. Am. Chem. Soc. 68, 1704 (1946).

3 M. Oguni, H. Hikawa, and H. Suga, Thermochim. Acta 158, 143 (1990).

4 O. Haida, H. Suga and S. Seki, J. Chem. Thermodyn. 9, 1113 (1979).

5 M. Sugisaki, H. Suga and S. Seki, Bull. Chem. Soc. Jpn. 41, 2586, 2591 (1968).

6 E. J. Sare, unpublished work.

7 J. F. Counsell, E. B. Lees, and J. F. Martin, J. Chem. Soc. (A), 1819 (1968) (Analysis by D. L. Smith).

8 (a) C. Alba, L. E. Busse, and C. A. Angell, J. Chem. Phys. 92, 617–624 (1990). (b) C. Alba-Simionesco, A. Vessal, J. Fan, and C. A. Angell, J. Chem. Phys. (in press).

9 C. A. Angell and D. L. Smith, J. Phys. Chem. 86, 3845 (1982).

10 K. Takeda, O. Yamanamuro, I. Tsukushi, and T. Matsui, Fluid Phase Equilibria (in press); private communication.

11 D. L. Smith, unpublished work.

12 G. E. Gibson and W. F. Giauque, J. Am. Chem. Soc. 45, 93 (1923).

13 J. E. Kunzler and W. F. Gianque, J. Am. Chem. Soc. 74, 797 (1952).

14 J. Fan, Ph.D. thesis, Arizona State Univ. (1995).

15 (a) H. Fujimori, M. Mizukami, and M. Oguni (to be published). (b) H. Fujimori, M. Oguni, and C. Alba-Simionesco, Proc. IUPAC Conference on Thermodynamics, Osaka, August 1996.

16 T. Hikima, M. Hanaya, and M. Oguni, Sol. State Comm. 93, 713 (1995).

17 W. T. Laughlin and D. R. Uhlmann, J. Phys. Chem. 76, 2317 (1972).

18 S. S. Chang and A. B. Bestul, J. Chem. Phys. 56, 503 (1972); R. J. Greet and D. Turnbull, J. Chem. Phys. 47, 2185 (1967).

19 H. Fujimori and M. Oguni, J. Phys. Chem. Sol. 54, 271 (1993).

20 H. Fujimori and M. Oguni, J. Chem. Thermodyn. 26, 367 (1994).

21 C. A. Angell and J. C. Tucker, J. Phys. Chem. 78, 278 (1974).

22 X. Yu and L. Heppler et al., J. Chem. Thermodyn. 25, 191 (1992).

23 W. Kauzmann, Chem. Rev. 43, 219 (1948).

24 W. Sichina and C. A. Angell, unpublished work.

25 Cited in Ref. 58.

26 S. S. Chang and A. B. Bestul, J. Chem. Thermodyn. 6, 325 (1974).

27 C. A. Angell, E. Williams, K. J. Rao, and J. C. Tucker, J. Phys. Chem. 81, 238 (1977).

28 Chap. 1 in Glass: Structure by Spectroscopy, J. Wong and C. A. Angell, eds., Marcel Dekker, New York, New York (1976).

29 C. A. Angell, C. A. Scamehorn, D. L. List, and J. Kieffer, eds., Proceedings of the XVth International Congress on Glass, O.V. Mazurin, Leningrad, NAVKA (1989) p. 204.

30 P. Richet, Geochim. Cosmochim. Acta. 48, 471 (1984).

31 Y. Takeda, O. Yamamuro, and H. Suga, J. Phys. Chem. Sol. 52, 607 (1991); M. Oguni (private communication).

32 *T*_0_ obtained from the slope of the *T*_g_-scaled Arrhenius plot, called fragility *m*, and its relation to *T*_0_ and *T*_g_. *T*_0_ = *T*_g_ (1 – 16/*m*) for relaxation times, which presumes log*τ*_0_ = 14, and *T*_0_ = *T*_g_ (1 – 17/*m*) for viscosity. See Roland Böhmer and C. A. Angell, Phys. Rev. B. 45, 10091 (1992). Many values of *m* are collected in R. Böhmer, K. L. Ngai, C. A. Angell and D. J. Plazek, J. Chem. Phys. 99, 4201 (1993).

33 A. C. Ling and J. E. Willard, J. Phys. Chem. 72, 1918 (1968); *m* = 58; also see Fig. 6 of Ref. 8. The value *T*_0_ = 59 K is reported for viscosity fits of the unbranched isomer hexane, by O. G. Lewis, J. Chem. Phys. 43, 2693 (1965).

34 B. Schiener, A. Loidl, R. V. Chamberlin, and R. Böhmer, J. Mol. Liq. 69, 243 (1996).

35 D. L. Smith and C. A. Angell, unpublished data on ethanol-methanol mixtures.

36 F. Stickel, E. W. Fischer, R. Richert, J. Chem. Phys. 104, 2043 (1996).

37 (a) D. L. Denney and R. H. Cole, J. Chem. Phys. 23, 1767 (1955) and D. L. Smith and C. A. Angell, unpublished data on ethanol-methanol mixtures. (b) N. Karger, T. Vardag, and H.-D. Lüdemann, J. Chem. Phys. 93, 3437 (1990).

38 D. W. Davidson and R. H. Cole, J. Chem. Phys. 19, 1484 (1951).

39 (a) D. B. Davies and A. J. Matheson, J. Chem. Phys. 45, 1000 (1966). (b) L. Wu, Phys. Rev. B 43, 9906 (1991).

40 B. Schiener and R. Böhmer, J. Non-Cryst. Sol. 182, 180 (1995).

41 From data tabulated in Ref. 9.

42 N. O. Birge, Phys. Rev. B 34, 1631 (1986).

43 S. Matsuoko, G. Williams, G. E. Johnson, E. W. Anderson, and T. Furukawa, Macromolecules 18, 2652 (1985).

44 I. M. Hodge, J. Non-Cryst. Sol. 169, 211 (1994); Table 2.

45 (a) F. Stickel, E. W. Fischer, A. Schönhals, and F. Kremer, Phys. Rev. Lett. 73, 2936 (1994). (b) H. Z. Cummins et al., Phys. Rev. Lett. 73, 2935 (1994).

46 E. J. Sare, Ph. D. thesis, Purdue University (1970).

47 Fujimori and M. Oguni (private communication).

48 P. K. Dixon, Phys. Rev. B. 42, 8179 (1990).

49 (a) P. K. Dixon and S. R. Nagel, Phys. Rev. Lett. 61, 341 (1988) (*o*-terphenyl + 9 %; *o*-phenylphenol, *m* = 81). (b) *T*_0_ = *T*_g_(1 – 16/*m*), see Ref. 32.

50 G. Williams and P. J. Hains, Faraday Symp. Chem. Soc. No. 6, 14 (1972).

51 A. J. Barlow, J. Lamb, and A. J. Matheson, Proc. Roy. Soc. A 292, 322 (1966).

52 (a) A. Schönhals F. Kremer, and H. Finch, Physica A201, 263 (1993). (b) C. A. Angell, L. Boehm, M. Oguni, and D. L. Smith, J. Mol. Liquids 56, 275–286 (1993).

53 C. T. Moynihan, J. Phys. Chem. 70, 3399 (1966).

54 J. H. Ambrus, C. T. Moynihan, and P. B. Macedo, J. Electrochem Soc. 119, 192 (1972).

55 C. A. Angell and R. D. Bressel. J. Phys. Chem. 76, 3244 (1972).

56 C. T. Moynihan, C. R. Smalley, C. A. Angell, and E. J. Sare, J. Phys. Chem. 73(6), 2287 (1969).

57 E. Sanchez and C. A. Angell (to be published).

58 A. B. Bestul and S. S. Chang, in Proceedings III International Congress on Glass, Venezia, 1953, A. Garzanti, ed., Nello Stabilimento Grafico Di Roma Della, Rome (1954) p. 26.

59 C. A. Angell, R. Stell, and W. J. Sichina, J. Phys. Chem. 86, 1540 (1982).

60 A. J. Easteal and C. A. Angell, J. Chem. Phys. 56, 4231 (1972).

61 C. A. Angell and W. Sichina, Ann. N.Y. Acad. Sci. Vol. 279 (1976) p. 53, for fragility (D) and Ref. 32.

62 P. Richet and Y. Bottinga, Earth Plan. Sci. Lett. 67, 415 (1984).

63 M. Tatsumisago, B. L. Halfpap, J. L. Green, S. M. Lindsay, and C. A. Angell, Phys. Rev. Lett. 64, 1549 (1990).

64 G. Fytas, G. N. Papatheodorou and E. A. Pavlatou, see Ref. 32.

65 M. Goldstein and M. Nakonecjnyi, Phys. Chem. Glasses 6, 126 (1965).
